# Supplementary material for: Different pedagogical approaches to motor imagery both demonstrate individualized movement patterns to achieve improved performance outcomes when learning a complex motor skill
Source: PLoS One. 2023 Nov 29;18(11):e0282647. doi: 10.1371/journal.pone.0282647 (PMC10686457; doi:10.1371/journal.pone.0282647)
Supplement: S1 File — (PDF) [file pone.0282647.s001.pdf]

Motor imagery instructions for NLP and LP conditions

LP condition

Welcome to your 4-week power clean training program. You will complete 3 sets of 5 repetitions of the power clean movement with 2-5minutes rest in between each set.

Get ready for ready for your first set

Imagine yourself in the set-up position… Bring your attention to what you see in the room around you. Find a point in the room to focus your attention throughout the duration of this set. As you set up you move your feet to hip width apart with your toes slightly turned out. Feel the tension in your muscles as you are in a squat position….. feel the rough grip of the bar as it sits in your hands. You grip the bar with your hands slightly wider than shoulder width apart.

Feel the muscles in your body tighten as you prepare to pull the bar. As you pull the bar from the floor your shoulders are over the bar, eyes looking forward and the bar is close to your shins. Your hips and knees are extending and the angle of your torso stays the same keeping your hips from rising too quickly.

As the bar passes your knees you push your knees forward keeping the bar in contact with your thighs, in this position you forcefully extend the hips, knees and ankles upwards contacting the bar with your hips.

As your lower body extends you explosively shrug your shoulders, pointing your elbows straight upwards.

As you reach the top of your pull your arms flex your elbows rotating your arms and hands as you pull your body under the bar. As you catch the bar your hips and knees are flexed, your head is facing forwards, back neutral, feet flat on the floor with your body weight resting over the middle of your feet.

You have completed your first repetition! You have 4 more repetitions to go.

In your own time complete the remaining 4 repetitions and signal to the coach once you have completed the remaining repetitions.

NLP condition (No constraints)

Welcome to your 4-week power clean training program. You will complete 3 sets of 5 repetitions of the power clean movement with 2-5minutes rest in between each set.

Get ready for ready for your first set

Imagine yourself in the set up position…. Bring your attention to what you see in the room around you. Find a point in the room to focus your attention throughout the duration of this set. Feel the tension in your muscles as begin your set up. As you set up think about leaning forward like you are just about to sit on a chair. As you set up and reach down to hold the bar… feel the rough grip of the bar as it sits in your hand. At this point think about staying connected to the bar keeping it as close and connected to your body throughout the movement.

Feel the muscles in your body tighten as you prepare to pull the bar. As you begin to pull the bar from the floor you feel your body become firm like a steel rod. As the bar moves from the floor you feel the bar is staying connected to your body as you pull the bar upwards. At this point you are moving upwards and the bar is moving like a train on a track staying close to the body and moving in the shape of a hook. You are connected to the bar pulling straight up like you are pulling your pants up and giving yourself a wedgie. As the bar moves you flick the bottom of your shirt, from here you explode jumping straight up. You pull the bar upwards like you are throwing it into the roof as the bar comes down in rests on your shoulders which act like a shelf for the bar to rest on.

You have completed your first repetition! You have 4 more repetitions to go.

In your own time complete the remaining 4 repetitions and signal to the coach once you have completed the remaining repetitions.

NLP condition (Constraints)

Welcome to your 4-week power clean training program. You will complete 3 sets of 5 repetitions of the power clean movement with 2-5minutes rest in between each set.

Get ready for ready for your first set

To begin your set up think about leaning forward like you are just about to sit on a chair. As you set up and reach down to hold the bar think about staying connected to the bar keeping it as close and connected to your body throughout the movement. Imagine yourself in the set up position… Bring what you see in the room around you. Find a point in the room to focus your attention throughout the duration of this set Feel the rough grip of the bar as it sits in your hands. Now bring your attention to the two poles on your left and right side placed in front of the bar. Throughout lift avoid hitting the poles with the bar. Still in the set-up position become aware of the chalk that is on the bar. As you lift leave a chalk mark from just above your knee to the top of your thigh.

As you begin to pull upwards avoid hitting the two poles that are on your left and right, as the bar continues to move upward keep it connected to your body. As the bar continues to move upwards think about the bar following a train track staying close to the body, aiming to move the bar in the shape of a hook. At this point pull the bar upward the same way you would pull up a pair of pants, as you do this become aware of the bar contacting the body and leaving a chalk mark on your pants as you pull the bar upwards. Staying connected to the bar try and flick the bottom of your shirt as you pull the bar upwards. You now explode upwards like you are jumping straight up, throwing the bar into the roof. The bar finishes on your shoulders like a shelf for the bar to rest on.

You have completed your first repetition! You have 4 more repetitions to go.

In your own time complete the remaining 4 repetitions. Once you have completed each repetition signal to the coach and proceed to the next repetition.
